# Evaluation of High-Temperature and Low-Temperature Performances of Lignin–Waste Engine Oil Modified Asphalt Binder and Its Mixture

**DOI:** 10.3390/ma15010052

**Published:** 2021-12-22

**Authors:** Xue Xue, Junfeng Gao, Jiaqing Wang, Yujing Chen

**Affiliations:** 1 Key Laboratory of Transport Industry of Road Structure and Materials (Xi’an), Chang’an University, Xi’an 710064, China; xxue@chd.edu.cn (X.X.); yujingchen90@163.com (Y.C.); 2 Xi’an Municipal Engineering Design & Research Institute Co., Ltd., Xi’an 710065, China; 3 National & Local Joint Engineering Laboratory of Transportation and Civil Engineering Materials, Chongqing Jiaotong University, Chongqing 400074, China; 4 College of Civil Engineering, Nanjing Forestry University, Nanjing 210037, China

**Keywords:** lignin, waste engine oil, modified asphalt, high-temperature performance, low-temperature performance

## Abstract

This research aims to explore the high-temperature and low-temperature performances of lignin–waste engine oil-modified asphalt binder and its mixture. For this research, the lignin with two contents (4%, 6%) and waste engine oil with two contents (3%, 5%) were adopted to modify the control asphalt binder (PG 58-28). The high-temperature rheological properties of the lignin–waste engine oil-modified asphalt binder were investigated by the viscosity obtained by the Brookfield viscometer and the temperature sweep test by the dynamic shear rheometer. The low-temperature rheological property of the lignin–waste engine oil-modified asphalt binder was evaluated by the stiffness and m-value at two different temperatures (−18 °C, −12 °C) obtained by the bending beam rheometer. The high-temperature and the low-temperature performances of the lignin–waste engine oil-modified asphalt mixture were explored by the rutting test and low-temperature bending beam test. The results displayed that the rotational viscosity and rutting factor improved with the addition of lignin and decreased with the incorporation of waste engine oil. Adding the lignin into the control asphalt binder enhanced the elastic component while adding the waste engine oil lowered the elastic component of the asphalt binder. The stiffness of asphalt binder LO60 could not meet the requirement in the specification, but the waste engine oil made it reach the requirement based on the bending beam rheometer test. The waste engine oil could enhance the low-temperature performance. The dynamic stabilities of LO40- and LO60-modified asphalt mixture increased by about 9.05% and 17.41%, compared to the control mixture, respectively. The maximum tensile strain of LO45 and LO65 increased by 16.39% and 25.28% compared to that of LO40 and LO60, respectively. The high- and low-temperature performances of the lignin–waste engine oil-modified asphalt LO65 was higher than that of the control asphalt. The dynamic stability had a good linear relationship with viscosity, the rutting factor of the unaged at 58 °C, and the rutting factor of the aged at 58 °C, while the maximum tensile strain had a good linear relationship with m-value at −18 °C. This research provides a theoretical basis for the further applications of lignin–waste engine oil-modified asphalt.

## 1. Introduction

The vigorous development of road construction and maintenance, the continuous increase in traffic volume, and the continuous changes of the local climate and environment in China have necessitated higher requirements for the performance of various aspects of asphalt pavement materials [1,2]. In order to improve the overall performance of asphalt materials, different chemical or physical modifiers are used to add to the base asphalt [3,4,5,6]. In recent years, some waste recyclable materials [7,8], especially the biomass materials [9,10,11], have been introduced into the road engineering field as a good modifier, and they have received more and more attention. The use of waste and recyclable materials can improve the performance of asphalt materials, promote the recycling of waste, and reduce the pollution caused by its burial and disposal, which has good application prospects [12]. 

Among the waste and recyclable materials, lignin has attracted many researchers. As a by-product or waste material of biomass materials, lignin is used as a modifier of asphalt materials and has great potential performance. It is a type of complex organic polymer, which forms important structural materials in the supporting tissues of vascular plants and some algae [13]. Among woody plants, lignin accounts for 25%, making it the second most abundant organic matter in the world. Lignin is a biopolymer with a three-dimensional network structure formed by connecting three kinds of phenylpropane units through ether bonds and carbon-carbon bonds [14,15,16]. It is rich in aromatic ring structures, aliphatic and aromatic hydroxyl groups, and active groups (such as quinone groups). In the field of papermaking, lots of lignin by-products are (about 50 million tons) produced, which are directly discharged into natural water in the form of pollutant “black liquor” [17]. Meanwhile, less than 20% of the lignin can be effectively used because of the concentration and burning of the byproducts [18]. Thus, less lignin can be used for resource utilization, resulting in a waste of organic resources.

In recent years, according to the properties and application status of lignin, researchers in the road field have carried out research on the preparation of modified petroleum-based asphalt by combining lignin and asphalt binder. Gao et al. introduced lignin as a biological additive to modify virgin asphalt, and studied the high-temperature rheological properties of lignin-modified asphalt and base asphalt [19]. The results displayed that the activation energy had an increasing trend with the increase in the lignin compared to the virgin asphalt. When the content of lignin was less than 8%, the reduction in fatigue life of lignin modified asphalt binder was small. Pan took biopolymers, namely coniferol lignin, as an example, and studied the chemical and physical basis of general chemical antioxidants [18]. He also used the X-ray photoelectron spectroscopy (XPS) technology to prove the validity of the lignin-modified and unmodified asphalt models, and the XPS results were highly consistent with the model predictions. Batista et al. investigated the physical and chemical properties of lignin-modified asphalt binder, and explored the feasibility for the applying of the lignin from the pulp and paper industry to the asphalt pavements [20]. The results revealed that the lignin-modified asphalt had low carbonyl index, which was beneficial to the weather aging resistance. Xie et al. proved the potential of lignin as an asphalt binder modifier by deriving the lignin fractions through enzyme-mediator-based biological processing and formic acid-based chemical processing [21]. Arafat et al. used three different types of lignin (kraft paper lignin, black liquor precipitation lignin, laboratory lignin produced from rice husks using a deep eutectic solvent) to replace a small portion (up to 6%) of asphalt to determine the effect of lignin on the aged and unaged asphalt binders and mixtures [22]. It was concluded that 6% of base asphalt binder can be replaced by the lignin from agricultural waste.

At the same time, waste engine oil is the non-distillable part collected from the solid-liquid copolymer formed from engine oil after being consumed by oxidation, etc. [23]. Waste engine oil and asphalt have relatively similar components and good recyclability value. In recent years, waste engine oil and its extracts have been regarded as good asphalt modifiers. Some researchers in the field of road pavement have delved into these aspects. Liu et al. used 4 wt% and 8 wt% waste engine oil to modify the asphalt binder from three different sources, and analyzed the chemical composition, molecular weight, and rheological properties [24]. They concluded that waste engine oil significantly reduced the viscosity of the adhesive and lowered the application temperature of asphalt binder. They also evaluated the aging behavior of waste engine oil-modified asphalt binder through testing the conventional performance, rheological properties, and micro characteristics of the aging residue [25]. Hesp et al. recorded the waste oil residues found on the pavement in Ontario and Canada, and inferred that the use of waste oil residues in asphalt was common based on zinc dialkyldithiophosphate as a general additive in engine oil. They also found that typical modification levels are in the range of 5–20% based on the XRF analysis of pure waste oil residues [26]. Li et al. evaluated a limited but well-controlled set of asphalt binders with the same performance level containing the recycled engine oil residues by the content of 0%, 2.5%, 6%, and 15% [27]. It observed that the fatigue cracking performance of the recycled engine oil residues at moderate temperature depended on the aging conditions and a stress/strain control model.

Based on the previous literature review, it can be observed that most of the researches focused on the lignin or waste engine oil, and the single additive adding into the asphalt binder could improve one aspect of the property of asphalt and decreased other properties. Meanwhile, there is rarely information about the performances of lignin–waste engine oil-modified asphalt from the perspective of binder and mixture. The relationship between the indexes of binder and indicators of lignin–waste engine oil-modified asphalt mixture was also rarely investigated. Thus, this research aims to add both the lignin and waste engine oil into the base asphalt to modify it, and explore the high-temperature and low-temperature performance of lignin–waste engine oil-modified asphalt binder and its mixture based on the Brookfield viscometer, dynamic shear rheometer, bending beam rheometer, the rutting test, and the low-temperature bending beam test.

## 2. Materials and Methods

### 2.1. Materials

#### 2.1.1. Base Asphalt

The base asphalt utilized was PG 58-28, which was regarded as the control in the experiments. The specific gravity was 1.03. The properties of this base asphalt met the requirements in the specification.

#### 2.1.2. Lignin

The lignin used in this study was provided by Shanghai Jack Gump Industrial Co. Ltd (Shanghai, China). It was obtained from the waste wood chips. The carbon, hydrogen, and oxygen of this kind of lignin were about 64.2%, 5.8%, and 29.2%, respectively. 

#### 2.1.3. Waste Engine Oil

The waste engine oil was replaced from the engine oil of the car by the car repairing shop. It was black and brown. The viscosity was 50 mPa∙s, while the specific gravity was 0.91.

#### 2.1.4. Aggregate and Filler

The aggregates were limestone and the filler was powdered from the limestone. The properties of the coarse aggregate and fine aggregate met the requirements in the specification [28].

### 2.2. Preparation of Lignin–Waste Engine Oil-Modified Asphalt

To prepare the lignin–waste engine oil-modified asphalt, the base asphalt binder contained by a container was firstly heated to flow in the heating oven at about 160 °C. Then, the weighted lignin was added into the base asphalt by different dosages and they were placed for the shearing by the high-speed shear mixer. The shear speed, shear time, and shear temperature were 6000 rpm, 40 min, and 160 °C, respectively. After that, the prepared waste engine oil with different contents were slowly added into the mixed asphalt binders and stirred for about 30 min to make the mixture evenly mixed. The dosages of lignin used in this research were 4% and 6% while the contents of waste engine oil utilized were 3% and 5% based on the previous studies [19,23]. To conduct experiments clearly and efficiently, the modified asphalt binder with m% lignin and n% waste engine oil was denoted as LOmn. For instance, LO43 refers to the modified asphalt with 4% content of lignin and 3% of waste engine oil, while LO60 refers to the modified asphalt with 6% content of lignin and 0% of waste engine oil. In addition, PG 58-28 (Shell Bitumen China Solutions Centre, Shanghai, China) was used as the control for comparing.

### 2.3. Mixture Gradation and Preparation of Lignin–Waste Engine Oil-Modified Asphalt Mixture

To investigate the high-temperature and low-temperature performances of the lignin–waste engine oil-modified asphalt mixture, the gradation of AC-16 was selected for the asphalt mixture, as shown in Table 1. The optimum asphalt was 4.5%, which was determined according to the Marshall test in the specification [28]. The slab specimen was formed to conduct the rutting test; it was also cut further for the low-temperature bending beam test.

### 2.4. Test Methods

#### 2.4.1. Viscosity Test

The viscosity was tested by the Brookfield viscometer. Four temperatures (110, 135, 150, and 165 °C) and three rotational speeds (10, 20, and 50 rpm) were selected to test the viscosities of the lignin and waste engine oil-modified asphalt.

#### 2.4.2. Temperature Sweep Test

The temperature sweep test of the lignin–waste engine oil-modified asphalt binder was conducted by the dynamic shear rheometer. The unaged and rolling thin film oven (RTFO) aged-modified asphalt binders were used for the temperature sweep test. The diameter and spacing for the test sample were 25 mm and 1 mm, respectively. In this study, the temperatures were 52, 58, 64, 70, 76, and 82 °C, while the test frequency was 1.59 Hz. The phase angle and rutting factors obtained from the dynamic shear rheometer were used to analyze the high properties of the lignin–waste engine oil-modified asphalt binder.

#### 2.4.3. Bending Beam Rheometer Test

The low-temperature property of asphalt binder was tested with the bending beam rheometer (BBR) test at different temperatures. The bending beam rheometer test provides a measure of low-temperature stiffness and relaxation properties of asphalt binders. These parameters give an indication of an asphalt binder’s ability to resist low-temperature cracking. The asphalt for this test was firstly aged by the rolling thin film oven (RTFO) and pressure aging vessel (PAV) test. The bending creep stiffness modulus (S) and the slope of the creep curve (m) obtained from this test were utilized to evaluate the flexibility of asphalt and the ability to resist the crack under the low temperature. In this research, two temperatures, −18 °C and −12 °C, were selected based on the performance grade of the control asphalt.

#### 2.4.4. Rutting Test for Mixture

To evaluate the high-temperature performance of the mixture, a rutting test was conducted. The formed slab specimen was placed in the chamber for wheel tracking at 60 °C. The dynamic stability (DS) and rutting depth at 60 min obtained by the wheel tracker were analyzed to investigate the high-temperature performance of the lignin–waste engine oil-modified asphalt mixture. The detailed process was shown in the specification Test Methods of Bitumen and Bituminous Mixture for Highway Engineering (JTG E20-2011) [29].

#### 2.4.5. Low-Temperature Bending Beam Test

The low-temperature bending beam test was used to investigate the low-temperature performance of mixture. The size of the beam was 250 mm × 30 mm × 35 mm while the temperature was −10 °C ± 0.5 °C. The maximum tensile strain and strength obtained was analyzed to evaluate the low-temperature performance of the lignin–waste engine oil-modified asphalt mixture. The detailed process was shown in the specification Test Methods of Bitumen and Bituminous Mixture for Highway Engineering (JTG E20-2011) [29].

**Figure 1:**
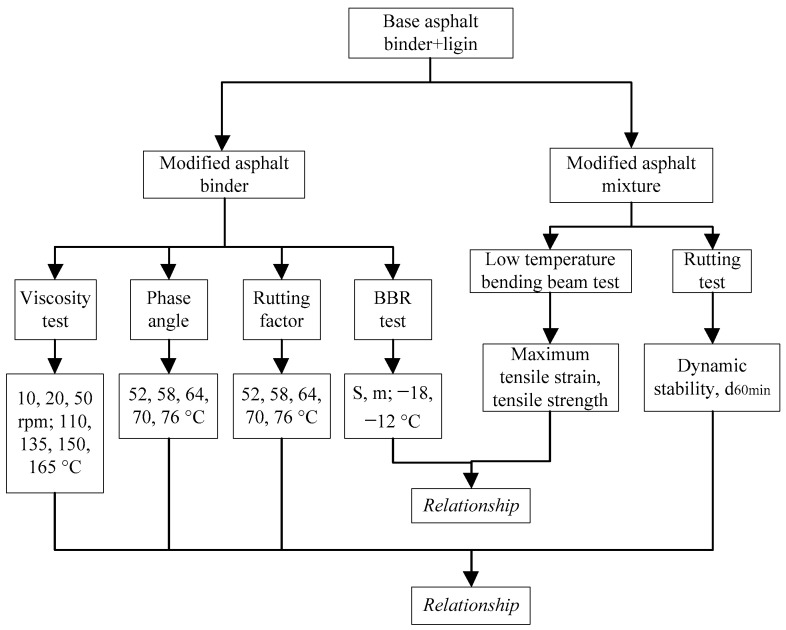
The map of this study.

The map of this study is shown in Figure 1.

## 3. Results and Discussion

### 3.1. Viscosity

**Figure 2:**
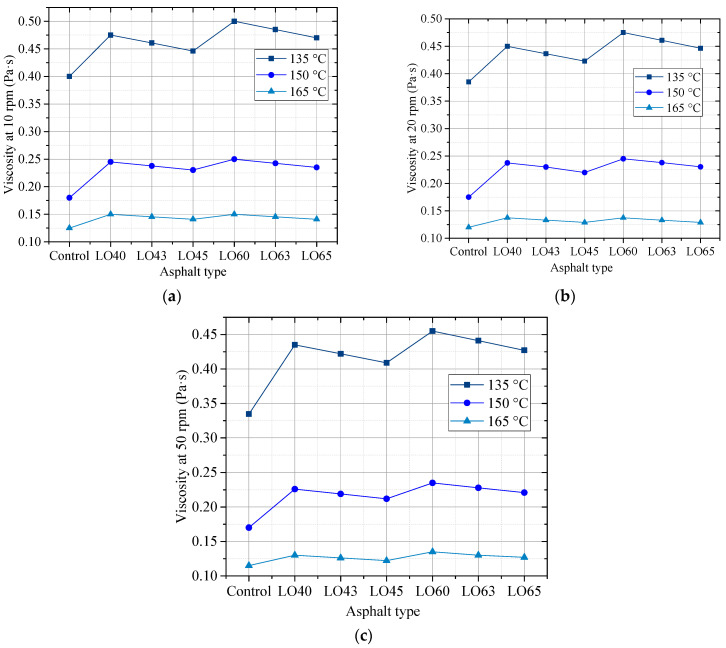
The viscosity of different asphalt types at different rotational speeds. (**a**) 10 rpm; (**b**) 20 rpm; (**c**) 50 rpm.

Viscosity is related to the resistance to the deformation under different actions from the external. Figure 2a–c shows the viscosity of control (PG 58-28) asphalt binder and lignin–waste engine oil-modified asphalt binders under different rotational speeds and temperatures. 

It can be found from Figure 2 that the viscosities of the lignin–waste engine oil-modified asphalt binders at 135 °C were less than the requirement value, 3.00 Pa∙s, in the specification. Increased temperature led to the decreased viscosities of different asphalt binder types. The decrease in viscosity between 150 °C and 165 °C was lower than that of the viscosity between 135 °C and 150 °C, regardless of the modified asphalt binder types and the rotational speeds. For instance, at 50 rpm, the viscosities of LO45 at 135 °C, 150 °C, and 165 °C were 0.41 Pa∙s, 0.21 Pa∙s, and 0.12 Pa∙s, respectively. Furthermore, the decrease in viscosity from 150 °C to 165 °C was 0.09 Pa∙s, while the decrease from 135 °C to 150 °C was 0.20 Pa∙s. This meant that the temperature had a significant influence on the viscosity. Take the asphalt binder control, LO40 and LO60 to analyze, it can be concluded that the addition of lignin increased the viscosity of asphalt binder, regardless of the rotational speeds. However, compared to LO40, the viscosities of LO43 and LO45 decreased, and the viscosity of LO45 decreased more than that of LO43. The viscosities of LO63 and LO65 showed the same change rule with that of LO43 and LO45. This indicated that the incorporation of waste engine oil decreased the viscosity of base asphalt binder and modified asphalt binder. This may be caused by the light component existing in the waste engine oil.

**Figure 3:**
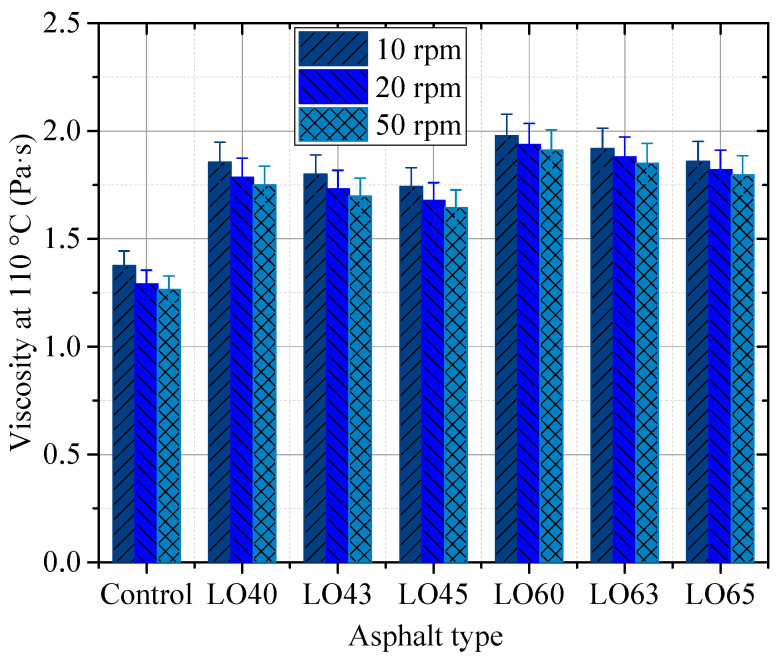
The viscosity of different asphalt types at 110 °C.

Figure 3 displays the viscosity of the control asphalt binder and the lignin–waste engine oil-modified asphalt binder at 110 °C. As shown in Figure 3, the addition of lignin increased the viscosity of asphalt binder, while the incorporation of waste engine oil decreased the viscosity of the lignin-modified asphalt binder. In addition, with the increase in the rotational speed, the viscosity decreased, regardless of the asphalt types at 110 °C. This indicated that the lignin–waste engine oil asphalt binders were non-Newtonian fluid, following the previous findings on the asphalt binder.

### 3.2. Temperature Sweep

#### 3.2.1. Phase Angle

**Figure 4:**
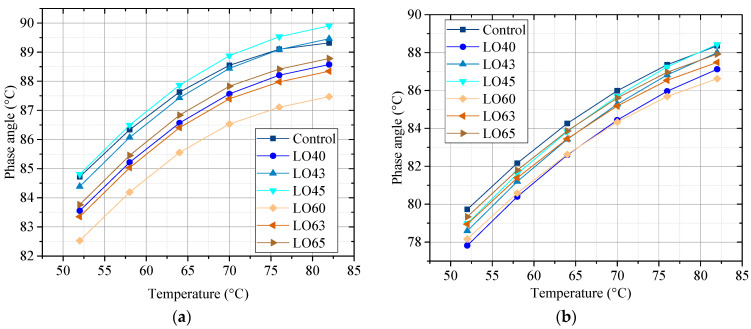
Phase angle of the unaged and the RTFO-aged asphalt binders. (**a**) The unaged; (**b**) The RTFO-aged.

Figure 4a,b shows the phase angle of the unaged and RTFO-aged control asphalt binder and the lignin–waste engine oil-modified asphalt binder.

The phase angle reflects the viscoelasticity of the asphalt binder. The smaller the phase angle, the higher the elastic part. It can be seen from Figure 4 that the phase angles of the control asphalt binder and lignin–waste engine oil asphalt binder increased with the increase in the temperature. This indicates that high temperatures give the asphalt binder less elastic portion. For the unaged asphalt binder, compared to the control, the phase angles of asphalt binder LO40 and LO60 decreased regardless of the test temperature, illustrating the improvement in the elastic portion by the addition of lignin. The phase angles of asphalt binder LO43 and LO45 increased compared to LO40, while the phase angles of LO63 and LO65 also enhanced compared to LO60. This meant that the incorporation of waste engine oil could improve the viscous component of the asphalt. Take the phase angles at 58 °C as an example, for the unaged asphalt, the phase angles of control, LO40, LO43, LO45, LO60, LO63, an dLO65 were 86.34°, 85.22°, 86.07°, 86.50°, 84.19°, 85.03°, and 85.45°, respectively. For the RTFO-aged asphalt binders, the phase angle of the control was higher than that of other asphalt binders, while the phase angle of asphalt binder LO40 and LO60 was lower than that of other asphalt binders. This was because the RTFO aging of lignin made the asphalt harder, and the more content of lignin, the obvious of this results. On the contrary, the waste engine oil could make the asphalt binder softer, leading to the difference of phase angle to the lignin-modified asphalt without waste engine oil.

#### 3.2.2. Rutting Factor 

**Figure 5:**
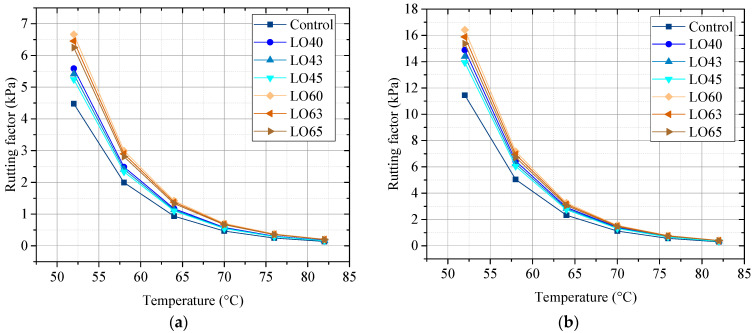
Rutting factor of the unaged and the RTFO-aged asphalt binders. (**a**) The unaged; (**b**) The RTFO-aged.

The rutting factor of the unaged and RTFO-aged control asphalt binder and lignin–waste engine oil-modified asphalt binder is shown in Figure 5a,b.

Rutting factor is used to evaluate the high property of the asphalt binder. As shown in Figure 5, the rutting factors of lignin–waste engine oil-modified asphalt dramatically decreased with the increase in temperature from 52 °C to 64 °C, while it slowly decreased with the changes in temperature from 64 °C to 82 °C. The temperature had a significant influence on rutting factor, and the higher temperature lead to a lower rutting factor, indicating lower high property of asphalt binder. For the unaged, the rutting factor of asphalt binder could be divided to three groups: the control, the LO4 group (LO40, LO43, LO45), and the LO6 group (LO60, LO63, LO65). Furthermore, it can be seen as obvious that the rutting factor of group LO6 was higher than that of group LO4, and that the rutting factor of the control was the lowest. This demonstrated that the incorporation of lignin could improve the high property of asphalt binder. Within the group of LO4, the rutting factor of LO40 was the highest, followed by LO43 and LO45, indicating the influence of waste engine oil on the lignin modified asphalt binder. The rutting factor within the group of LO6 had the same changes. For instance, when the temperature was 58 °C, the rutting factors of LO60, LO63, and LO65 were 2.995 kPa, 2.901 kPa, and 2.809 kPa, respectively. For the RTFO-aged, the rutting factors of different lignin–waste engine oil-modified asphalt binder displayed the same change as the unaged, though the group was not divided.

### 3.3. Bending Beam Rheometer Test

**Figure 6:**
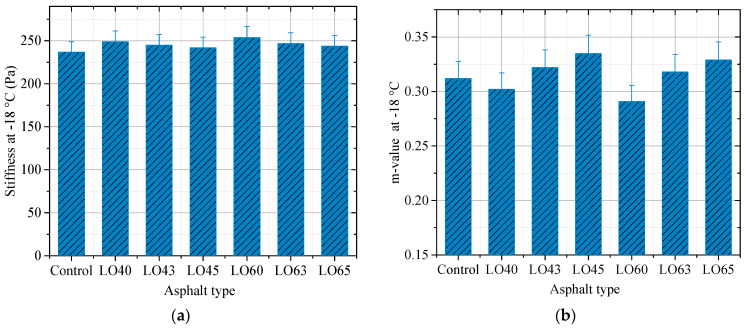
The BBR results at −18 °C. (**a**) Stiffness; (**b**) m-value.

**Figure 7:**
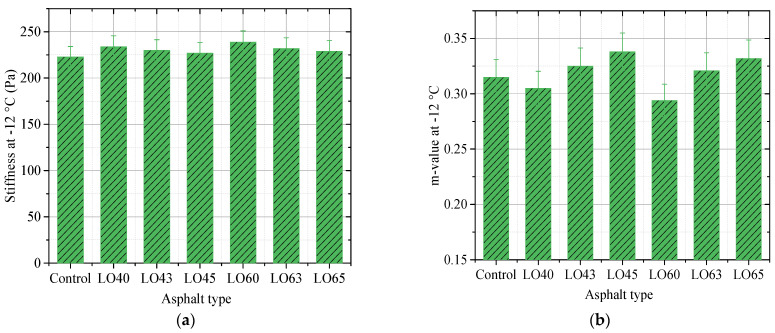
The BBR results at −12 °C. (**a**) Stiffness; (**b**) m-value.

The stiffness and m-value of the control and lignin–waste engine oil-modified asphalt binder obtained by the bending beam rheometer test are shown in Figure 6 and Figure 7.

It can be observed from Figure 6 and Figure 7 that a higher test temperature led to a lower stiffness. For example, the stiffness of asphalt binder LO63 at −18 °C and −12 °C were 247 Pa and 232 Pa, respectively. This could provide evidence for the utilized temperature condition of the modified asphalt binder. Besides, take the stiffness of the lignin–waste engine oil-modified asphalt binder at −18 °C as an example, all the results could meet the requirement for the using of asphalt binder in the specification, which is 300 Pa. The stiffness of asphalt binder LO40 and LO60 were 249 Pa and 254 Pa, respectively, which increased by about 5.1% and 7.2% compared to that of the control asphalt binder, respectively. Moreover, the stiffness of asphalt binder LO45 and LO65 decreased by 2.8% and 3.9% compared to that of LO40 and LO60, respectively. This indicated that the addition of lignin made the stiffness higher, while the waste engine oil had the opposite influence. Thus, the waste engine oil was beneficial to the low-temperature resistance of asphalt binder. In addition, the m-value of different kinds of asphalt binders, except for LO60 at −18 °C, was greater than 0.3. This meant that the stiffness of asphalt binder LO60 could not meet the requirement in the specification, but the waste engine oil could make it reach the requirement. The stiffness and m-value of lignin–waste engine oil-modified asphalt binder at −12 °C showed the same change rule as them at −18 °C. This can be attributed to the alkylbenzenes, as well as linear alkanes contained in the waste engine oil, which could dissolve the asphalt binder [30].

### 3.4. Rutting Test

The dynamic stability (DS) is the number of wheel passing on the surface of the slab specimen when the rutting depth reaches 1 mm. In this study, to investigate the high-temperature performance of lignin–waste engine oil-modified asphalt mixture, the dynamic stability (DS) and rutting depth at 60 min were obtained and analyzed. 

**Figure 8:**
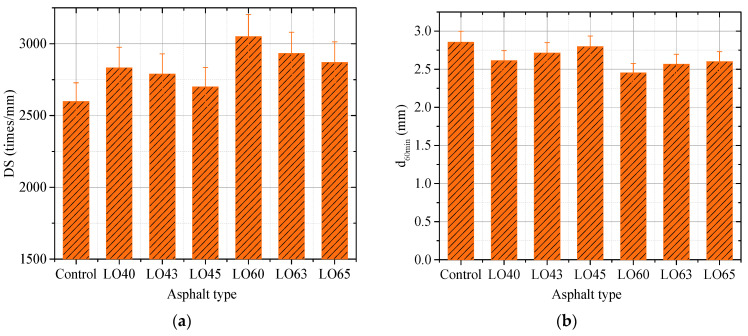
The dynamic stability and rutting depth at 60 min. (**a**) Dynamic stability (DS); (**b**) d_60min_.

Figure 8 illustrates the dynamic stability and rutting depth at 60 min of different lignin–waste engine oil-modified asphalt mixtures. As shown in Figure 8a, the dynamic stability of the control asphalt mixture was 2598.32 times/mm. The dynamic stabilities of LO40- and LO60-modified asphalt mixtures were 2833.56 times/mm and 3050.69 times/mm, which increased by about 9.05% and 17.41% compared to the control mixture, respectively. With the incorporation of waste engine oil, the dynamic stability decreased. For instance, the dynamic stability of LO43 was lower than that of LO40, followed by LO45. In addition, the dynamic stability of LO63 was lower than that of LO60, followed by LO65. However, the dynamic stability of the LO45 asphalt mixture was 2699.98 times/mm, which was still higher than that of the control. This illustrated that the addition of lignin could balance the attenuation of the high performance of asphalt mixture affected by the incorporation of waste engine. Moreover, the rutting depths at 60 min of different kind of asphalt mixtures from Figure 7b displayed opposite changes compared to the dynamic stability, illustrating the same change law as the dynamic stability Figure 8a. All the rutting depths at 60 min of the lignin–waste engine oil-modified asphalt mixture were higher than that of the control asphalt mixture. In all, the high-temperature performance of the lignin–waste engine oil-modified asphalt LO65 was higher than that of the control asphalt. This was in accordance with the findings by the rutting factor from the temperature sweep.

### 3.5. Low-Temperature Bending Beam Test

The maximum tensile strain and tensile strength obtained and calculated by the low-temperature bending beam test were used evaluate the low-temperature performance crack resistance of the asphalt mixture.

**Figure 9:**
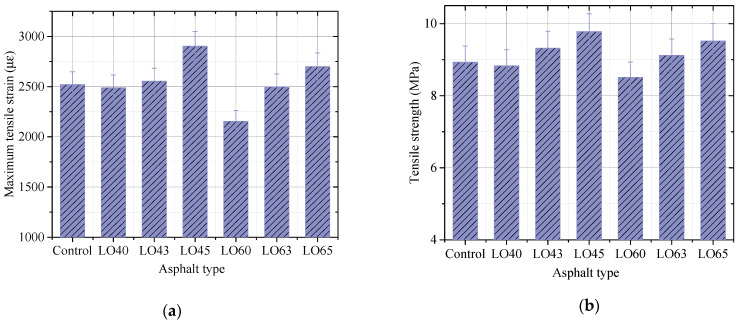
The maximum tensile strain and tensile strength. (**a**) Maximum tensile strain; (**b**) Tensile strength.

Figure 9 describes the maximum tensile strain and tensile strength of different kinds of lignin–waste engine oil-modified asphalt mixture. It can be investigated that the maximum tensile strain and tensile strength of LO60 asphalt mixture were 2155.61 με and 8.511 MPa, respectively, which were the lowest among the lignin–waste engine oil-modified asphalt mixture. The maximum tensile strain and tensile strength of LO40 asphalt mixture were higher than that of LO60, but they were also lower than other asphalt mixture. This indicated that the addition of lignin decreased the low-temperature resistance of asphalt mixture. Meanwhile, the waste engine oil increased the maximum tensile strain and tensile strength of LO40 and LO60. Take the maximum tensile strain as an example, the maximum tensile strain of LO45 and LO65 were 2903.55 and 2700.62, which increased by 16.39% and 25.28% compared to that of LO40 and LO60, respectively. Ultimately, the low-temperature performance of the lignin–waste engine oil-modified asphalt LO65 was higher than that of the control asphalt. This was consistent with the results of asphalt binder from the bending beam rheometer.

### 3.6. The Linear Fit of the Binder and Mixture

To analyze the relationship between the indexes of binder and indicators of the lignin–waste engine oil-modified asphalt mixture, the linear fit was conducted. 

**Figure 10:**
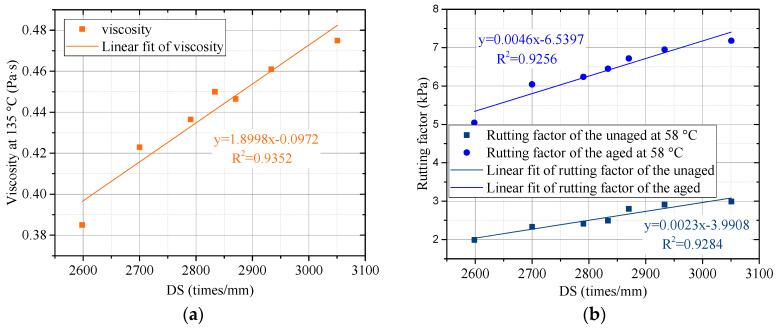
The linear fit of DS, viscosity, rutting factor. (**a**) DS with viscosity; (**b**) DS with rutting factor.

**Figure 11:**
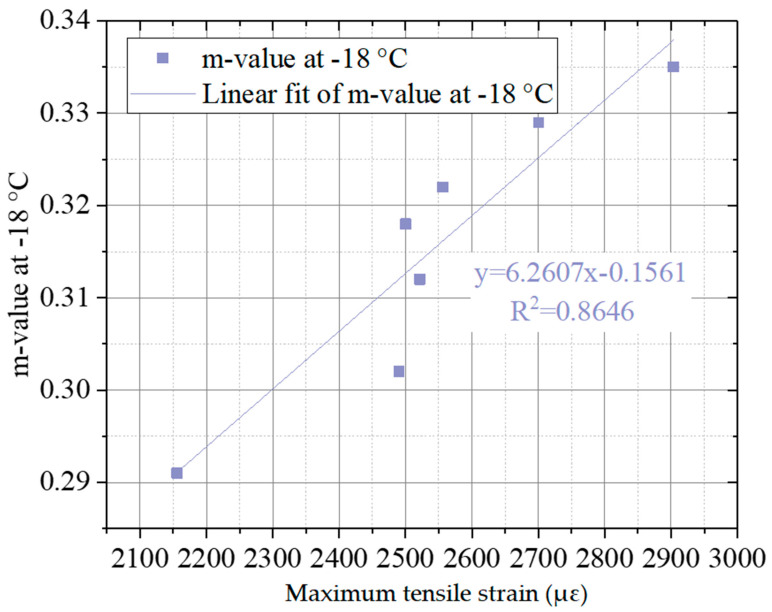
The linear fit of maximum tensile strain and m-value.

Figure 10a,b shows the linear fit of dynamic stability (DS) from the asphalt mixture, viscosity, and rutting factor, which indicated the high property of the lignin–waste engine oil-modified asphalt binder. The viscosity of different kinds of asphalt binders with 20 rpm at 135 °C was selected, and the rutting factors of the unaged and RTFO-aged asphalt binder at 58 °C were selected. It can be observed from Figure 10a,b that the R-square of dynamic stability and viscosity, the rutting factor of the unaged at 58 °C, and the rutting factor of the aged at 58 °C were 0.9352, 0.9284, and 0.9256, respectively. This indicated that the dynamic stability obtained by the asphalt mixture had a good linear relationship with viscosity, the rutting factor of the unaged at 58 °C, and the rutting factor of the aged at 58 °C, from the perspective of the high-temperature performance. The linear fit of maximum tensile strain and m-value is displayed in Figure 11. The m-value at −18 °C obtained by the bending beam rheometer test was selected. As shown in Figure 11, the R-square of maximum tensile strain and m-value was 0.8646, illustrating a good linear relationship between maximum tensile strain and m-value at −18 °C from the perspective of the low-temperature performance.

## 4. Conclusions

The high-temperature and low-temperature performances of lignin–waste engine oil-modified asphalt binder and its mixture were evaluated. The Brookfield viscometer, dynamic shear rheometer, and bending beam rheometer were utilized to test the properties of the asphalt binder, while the rutting test and low-temperature bending beam test were conducted to investigate the performances of the asphalt mixture. Based on the test results, the following conclusions can be drawn.

(1) The rotational viscosity improved with the addition of lignin and decreased with the incorporation of waste engine oil. Meanwhile, the viscosity of lignin–waste engine oil-modified asphalt can still meet the requirement for mixing and construction of asphalt binder in the specification.
(2) The rutting factors of lignin–waste engine oil-modified asphalt dramatically decreased with the increase in temperature from 52 °C to 64 °C, while it slowly decreased with the changes in temperature from 64 °C to 82 °C. Adding the lignin into the control asphalt binder enhanced the elastic component, while adding the waste engine oil lowered the elastic component of the asphalt binder.
(3) The stiffness of asphalt binder LO60 could not meet the requirement in the specification, but the waste engine oil helped it reach the requirement based on the bending beam rheometer test. The waste engine oil could enhance the low-temperature performance.
(4) The dynamic stabilities of LO40- and LO60-modified asphalt mixture increased compared to the control mixture, respectively. The maximum tensile strain of LO45 and LO65 increased compared to that of LO40 and LO60, respectively. The high- and low-temperature performances of the lignin–waste engine oil-modified asphalt LO65 was higher than that of the control asphalt.
(5) The dynamic stability obtained by the asphalt mixture had a good linear relationship with viscosity, a rutting factor of the unaged at 58 °C, and a rutting factor of the aged at 58 °C, from the perspective of a high-temperature performance. The maximum tensile strain and m-value at −18 °C had a good linear relationship from the perspective of a low-temperature performance.

Due to the limitation of experimental conditions of this study, the durability of the lignin–waste engine oil-modified asphalt was not studied. In the future, the comprehensive performances during the application in the field and the durability will be investigated.

## Figures and Tables

**Table 1:** The gradation of AC-16 in this research.

Sieve Size/mm	The Mass Percentage Passing through the Following Sieves/%										
	19	16	13.2	9.5	4.75	2.36	1.18	0.6	0.3	0.15	0.075
Composite gradation	100	95	88	75	47	32	23	17	12	8.5	6
Upper limit	100	100	92	80	62	48	36	26	18	14	8
Lower limit	100	90	76	60	34	20	13	9	7	5	4

## Data Availability

All data generated or analysed during this study are included in this published article.
